# The Association of Nutrition Quality with Frailty Syndrome among the Elderly

**DOI:** 10.3390/ijerph19063379

**Published:** 2022-03-13

**Authors:** Katarzyna Rolf, Aurelia Santoro, Morena Martucci, Barbara Pietruszka

**Affiliations:** 1 Department of Food Technology and Human Nutrition, University of Rzeszow, 35-601 Rzeszow, Poland; 2 Department of Experimental, Diagnostic and Specialty Medicine, University of Bologna, 40138 Bologna, Italy; aurelia.santoro@unibo.it (A.S.); morena.martucci3@unibo.it (M.M.); 3 Department of Human Nutrition, Warsaw University of Life Sciences—SGGW, 02-776 Warsaw, Poland; barbara_pietruszka@sggw.edu.pl

**Keywords:** elderly, diet, frailty, vitamin D, day care senior centers

## Abstract

Low diet quality among the elderly may be correlated with some diseases, including Frailty Syndrome (FS). This decline in function restricts the activity of older people, resulting in higher assistance costs. The aim of this study was to increase knowledge of diet quality predictors. Dietary intake was assessed among 196 individuals aged 60+ years using the three-day record method and FS by Fried’s criteria. Based on the compliance with the intake recommendation (% of EAR/AI), we distinguished three clusters that were homogeneous in terms of the nutritional quality of the diet, using Kohonen’s neural networks. The prevalence of frailty in the entire group was 3.1%, pre-frailty 38.8%, and non-frailty 58.1%. Cluster 1 (91 people with the lowest diet quality) was composed of a statistically significant higher number of the elderly attending day care centers (20.7%), frail (6.9%), pre-frail (51.7%), very low vitamin D intake (23.8% of AI), using sun cream during the summer months (always 19.8% or often 39.6%), having diabetes (20.7%), having leg pain when walking (43.1%), and deteriorating health during the last year (53.5%). The study suggests the need to take initiatives leading to the improvement of the diet of the elderly, especially in day care senior centers, where there are more frail individuals, including nutritional education for the elderly and their caregivers.

## 1. Introduction

Aging means a gradual, physiological, but not linear, decrease in physical and mental capacity. These changes are loosely associated with a person’s age in years; however, the elderly are usually classified as 60 years or older. Demographic statistics show that the world’s population is aging rapidly. It is estimated that the number of older people in the world will double by 2050 [1]. Therefore, a better understanding of all aspects of the aging process is needed, in particular with regard to the long-term care requirement for the elderly, which may generate additional social costs [2].

Aging is associated with many changes in the body’s cells, tissues, and organs, which may affect the functioning of, for example, the musculoskeletal and digestive systems, causing decreased food intake, as well as digestion and absorption of nutrients. The poor nutritional value of meals and the decreased ability of the digestive system contribute to the development of malnutrition. In addition, the elderly take drugs, such as diuretics, with potential adverse effects [3,4,5]. Many studies have shown that malnutrition and the risk of malnutrition concern from 25% to even above 60% of the population of older people [6,7,8,9]. The criteria for the diagnosis of clinical malnutrition are different in the USA and Europe. The lack of accurate criteria for diagnosing malnutrition increases the risk of adverse health outcomes [10,11]. The diet of seniors should take into account their lower energy requirements, while they have a greater need for proteins, vitamin D, and B6 as well as calcium. An adequate intake of folate, vitamin B12, and antioxidants is also important [3,12]. Therefore, a diet should have high nutritional density [3,13,14]. Age-related changes in protein metabolism mean that the average daily protein intake for the elderly should be at least 1.0 to 1.2 g per kilogram of body weight per day [12,13]. As the elderly exhibit decreased thirst sensation and reduced fluid intake, this results in dehydration, which is a form of malnutrition [14].

The aging process is often associated with Frailty Syndrome (FS); this means there is a weakening of the physical and/or mental condition, which in turn increases the risk of mortality [15,16]. One of the causes of FS may be malnutrition, especially a deficiency of energy and protein [17]. However, insufficient vitamin D status is also strongly associated with FS [18]. There is a correlation between FS and nutritional inadequacy [19]. Sarcopenia, i.e., the loss of muscle mass and strength progressing with age, is also associated with FS [20]. The frequency of the appearance of FS is higher among the elderly with sarcopenia [17]. It appears that higher protein intake by the elderly can be associated with a lower risk of FS [21] and sarcopenia [17].

Therefore, the aim of this study was to assess the relationship between the quality of nutrition and various determinants, including the FS, among individuals aged 60+ years.

## 2. Materials and Methods

### 2.1. Participants

The cross-sectional study was conducted at the Department of Human Nutrition, Warsaw University of Life Sciences—SGGW, Warsaw, Poland between 2012 and 2016. Independently-living participants aged 60 years and over were recruited. People applied for the study on the basis of information disseminated through leaflets, advertisements in the local press, as well as information provided in seniors’ clubs and the Universities of the Third Age. All respondents taking part in the study were volunteers. Some of them participated in the randomized and controlled NU-AGE project, previously described [22], and some were outside the project. Exclusion criteria were: 1. inability to perform daily activity (inability to walk), 2. advanced dementia (inability to sign informed consent), 3. failure to complete the three-day record, and 4. aged below 60 years. From the 229 recruited individuals, 33 did not fill in the three-day records, so 196 people were included in the study. 

The study protocol was approved on 3 April 2012 by the ethical commission of the National Food and Nutrition Institute in Warsaw, Poland. The study followed the principals of the Declaration of Helsinki.

### 2.2. Dietary Assessment

Dietary intake was assessed using the three-day record method, including one weekend day and two weekdays (nonconsecutive). Participants were instructed to take detailed notes on the food and beverages consumed, as well as the dietary supplement intakes, which were included in the analysis. Data on the amount of food consumed were collected using the weight method, and when it was not possible, the portion size was determined using home measures. The correctness of completing the questionnaires was verified by a qualified nutrition specialist. Based on the food consumption data, the nutrient content was calculated using the computer database ”Dieta 5”, which was based on Polish food composition tables [23]. The mean daily energy intake, as well as the intake of protein, fat, digestible carbohydrate, calcium, phosphorus, magnesium, iron, copper, zinc, iodine, vitamins A, B1, B2, B6, and B12, niacin, and folate was individually compared with the Estimated Average Requirements (EAR) for every respondent, while the consumption of water, dietary fiber, sodium, potassium, vitamin D and E, and also of eicosapentaenoic, docosahexaenoic, linoleic and alfa-linolenic fatty acids, was compared with the Adequate Intake (AI) [12].

### 2.3. Frailty Syndrome 

Frailty syndrome (FS) was evaluated according to the five criteria proposed by Fried et al. [15]: (1) unintentional weight loss (shrinking); (2) weakness; (3) exhaustion; (4) low walking speed; and (5) physical inactivity. Unintentional weight loss concerned a loss of more than 4.5 kg or 5% body weight in the previous year, based on self-reporting. Weakness was assessed as the mean value of three grip strength measurements using a handheld dynamometer (dominant hand). The results were considered separately for men and women, and additionally for BMI. Exhaustion was determined on the basis of the participants’ responses to two questions about how energetic and active they felt over the previous two weeks. Walking speed was measured over a distance of 4.5 m. The results were considered separately for men and women, and additionally for body height. The participants’ physical inactivity was determined on the basis of self-reported physical activity over previous year. In this case, participants answered how much sport or physical activity they usually did. Less than 2 h per week was considered as a criterion of frailty. Individuals were considered to be “frail” if they met ≥ 3 criteria, and those who had 1–2 criteria were classified as “pre-frail”. If participants did not have any of the described characteristics, they were categorized as “non-frail”. The original questionnaire was translated into Polish as part of the NU-AGE project.

### 2.4. Statistical Analysis

**Figure 1:**
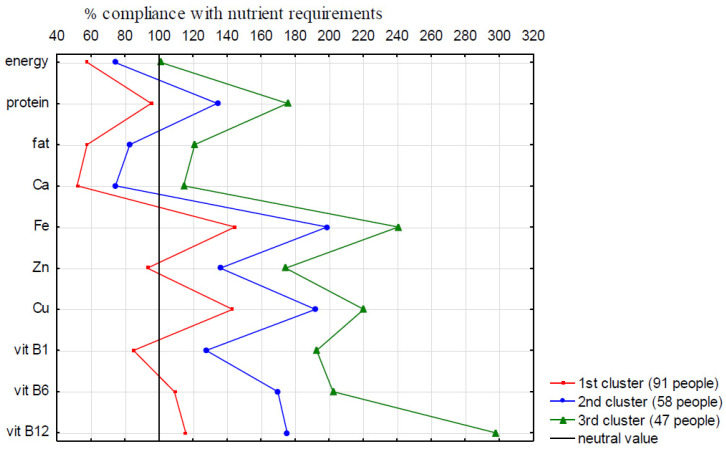
Division of the respondents into clusters, by the Kohonen’s neural networks.

Data were analyzed using Statistica software version 13.3 (TIBCO Software Inc., Palo Alto, CA, USA). Based on the comparison of the mean level of compliance with nutrient requirements (% of EAR or AI) between individuals depending on frailty status, we distinguished groups that were more homogeneous in terms of the nutritional quality of the diet, using the Kohonen’s neural networks (KNN). First, we performed the statistical analysis of association between FS and nutrients intake, using the Mann-Whitney U test. Energy intake, as well as intake of protein, fat, calcium, iron, zinc, copper, vitamins B1, B6, B12 were included in the KNN analysis, due to the statistically significant correlation with FS. The final number of clusters and their size were determined on the basis of the analysis of variance and the Pearson correlation coefficient between the clusters and nutrients included in the cluster analysis, as well as the correlation between the clusters and the prevalence of FS. In Figure 1, showing the division of population under study into 3 clusters, the lines connecting the points have been preserved due to better data visualization and easier location of individual points. There is also a “neutral value” line showing 100% of compliance with the recommended nutrient intake. The Pearson’s chi-squared test was used to determine the association between clusters and categorical variables; for continuous variables the Kruskal-Wallis test and post hoc analysis was used. The results with *p*-values ≤ 0.05 were considered statistically significant.

## 3. Results

Cluster analysis showed three clusters clearly differing in the degree of implementation of nutritional standards for energy, protein, fat, calcium, iron, copper, and vitamin B1, B6, and B12. The first cluster covered 91 people with nutritional standards on average below the EAR for six out of ten analyzed nutrients (energy, protein, fat, calcium, zinc, and vitamin B1). The second cluster included 58 people with the norms below the EAR for only two nutrients (fat and calcium), and the third one (47 people) with all nutrients above the EAR (Figure 1). Neither gender, age, marital status, education level, nor occupation were different among clusters. Over 20% of the individuals in cluster 1 attended day care senior centers, while in cluster 3 it was only about 2% (*p* = 0.0067) (Table 1). 

Although the total number of frail subjects was only six (3.1%) in the entire sample size, cluster 1 was characterized by a statistically significantly greater number of frail and pre-frail individuals, and fewer non-frail individuals (*p* = 0.0096). In individuals from cluster 1, weakness, exhaustion, and lower walking speed were observed much more often in comparison with the clusters 2 and 3 but no shrinking or physical activity (Table 2).

In the entire study group the majority of respondents reported their health as good (49.5%) or average (42.9%); however, in cluster 1 there were significantly more individuals who perceived a deterioration in health compared to the previous year (*p* = 0.0373; Table 2). Diabetes and leg pain during walking were observed significantly more often also among the elderly in cluster 1. Median BMI was the highest in cluster 1, but the difference with other clusters was not statistically significant (Table 2). 

We found statistically significant differences in summer sun exposure, sunscreen use, and vitamin D intake between the elderly from individual clusters (Table 3). In the first cluster, the fewest people declared no sun exposure in the year preceding the study (2.2%); simultaneously, they used sun cream the most during the summer months (39.6%) and had the lowest number of people who never used it (36.6%). Moreover, the consumption of vitamin D expressed as % of the AI norm was the lowest both with the diet alone (22% of AI) and after taking into account the consumption of this vitamin in the form of dietary supplements (23.8% of AI).

## 4. Discussion

Nutrition is considered to be one of the main factors in the pathophysiology of frailty syndrome (FS); therefore, proper nutrition is the goal of not only prevention but also treatment strategies [24]. Therefore, this cross-sectional study investigated whether malnutrition was related to those already confirmed to have frailty syndrome. 

Our survey was conducted among voluntarily enrolled participants, and recruitment took place, among others, at senior clubs and at Third Age Universities. Elderly people attending such institutions are more interested in a healthy lifestyle, do not have cognitive decline—which is associated with FS [25], and are more willing to participate in research, while weakened/frail elderly are usually less cooperative. The incidence of frailty syndrome in our study was low (3.1%) in comparison with the results of other studies, where the FS prevalence varied from below 10% [26,27], through over ten percent [25,28,29], to above 30% [19]. More frail people can be recruited in studies conducted in primary health care clinics, specialized outpatient clinics, or as a part of medical examinations, because there is a higher possibility to encounter such individuals [19,25]. Moreover, it is known that the average interest of frail people in taking part in research in such conditions is higher [30]. However, neither community-dwelling nor outpatient clinic recruitment would be representative, so the proportion of the frail elderly did not reflect the real number. 

The characteristics of the subjects were conducted within the division into three nutritional clusters. Among all the parameters presented in Table 1, a statistically significant relationship was found only for the parameter “Attending day care senior centers”. Among individuals attending this type of institution, the majority was in cluster 1. This is surprising, because in this type of senior center, the elderly receive meals. The reason for this should be further investigated, as our result may indicate some nutritional deficiencies in meals at such centers.

Our results showed that although a lack of association between age and nutritional quality of the diet was observed, the median age was a little higher among individuals with a poorer diet (cluster 1). Similarly, Engelheart and Akner [31] as well as Fakhruddin et al. [32] did not find correlation between age and food intake. However, in another Polish study, authors found a strong impact of age on poor nutritional status [33]. The difference between our study and the one quoted may be due to research methods. We analyzed the dietary intake, using the three-day record method, whereas Krzymińska-Siemaszko et al. [33] examined nutritional status, using the MNA questionnaire. The diet may not fully reflect the nutritional status, because, as mentioned earlier, it can be related to changes in the digestive system of the elderly [3,5].

Frailty syndrome can be related to the nutritional value of diets, especially as it can be the consequence of malnutrition or catabolism, which increase with age [15]. In our study, respondents with a low nutritional value diet were more likely to have at least one symptom of FS (frail and pre-frail). Several studies indicate a poorer diet in frail elderly people [27,34,35,36] and a generally lower density of nutrients in a diet [34]; however, no causal relationship data are available. Importantly, improving dietary habits reduces the odds of developing frailty [35]. However, it should be note that caloric/protein supplementation should be considered only for frail persons when weight loss or undernutrition is diagnosed [37]. On the other hand, research indicates that improving health status and extending life is associated with reducing the energy value of the diet, but with an adequate supply of macro-, and especially microelements, preventing malnutrition among healthy elderly [38]. Unfortunately, calorie restrictions survey are largely based on animals models. A nonhuman primate study showed that calorie reduction decreases the incidence of FS and increases the healthy life span [39].

In contrast to other studies [15,25,36], we found no differences between nutritional value of the diet (that is between clusters) in self-assessment of health. This could be due to the aforementioned form of recruitment elderly to the study. However, in cluster 1 there were significantly more people who reported a deterioration in health over the past year, as well as diabetics, and those who reported leg pain while walking, as in another study [15]. Taking into account that cluster 1 had the most people with FS, it can be concluded that there was an association between the occurrence of FS and these factors. Leg pain may accompany disability, which is strongly associated with FS [25], but may also contribute to the development of FS as an element of locomotive syndrome. Such people are at a higher risk of further deterioration of their health [40].

Among other analyzed factors related to diet quality, a statistically significant relationship was found with factors related to vitamin D metabolism. Vitamin D deficiency may affect up to 50% of the elderly population in Europe, therefore many countries have introduced mandatory fortification of some food products, for example, milk in Sweden and margarine in the United Kingdom [41]. In Poland, all table spreads, except milk fat, are mandatorily vitamin D fortified, so that the maximum amount in 100 g of the final product is not more than 7.5 μg [42]. 

It is worrying that almost 30% of our respondents avoided sunlight exposure during summer months, when endogenous vitamin D synthesis in Europe is the highest [43]. Although there were the least such individuals in cluster 1, that is, in the group with the highest percentage of frail and pre-frail people, in this cluster, there were a higher number of respondents using sun cream, which blocks vitamin D synthesis [43]. Moreover, vitamin D intake was very low in cluster 1, either with diet and supplements combined or with diet alone, as evidenced by only slightly higher median intake combined, compared to the diet only. It is equally important that synthesis of 1,25-dihydroxy-vitamin D decreases with age, among others reasons because of renal function decline [44]. As a consequence, the elderly are more likely to suffer from vitamin D deficiency, which may intensify FS symptoms. Wilhelm-Leen et al. [45], as well as Pabst et al. [46], found an inverse association between FS and 25-hydroxy-vitamin D serum concentration. One of the results of FS is an increased risk of falls [15]. In a randomized intervention study, ergocalciferol supplementation at an amount of 600,000 I.U. (15,000 µg) significantly improved functional performance, psychomotor skills, and balance in the elderly [47]. One of the mechanisms responsible for this may be the improvement in neuromuscular function by vitamin D [48], as atrophy in the first reacting fast-twitch type II muscle fibers has been observed in people with this deficiency, which may predispose them to more frequent falls and bone fractures [46]. Considering the above research, as well as Hirani et al. [18], confirming the benefits of a higher vitamin D level, it is worrying that in our study there were elderly with an intake at a level of 3% of AI norm, that is 0.45 µg (the Adequate Intake is 15 µg per day) [12]. Similarly, Verlaan et al. [17] found a very low intake of vitamin D in the entire study group, and among the elderly with sarcopenia the amount was much lower than among those without sarcopenia. Bartali et al. [34] also discovered a lower intake of this nutrient by frail elderly. 

One of the limitations of our study is its cross-sectional nature, which makes it impossible to assess the causal relationship between the intake of nutrients and analyzed factors, such FS. Another is the recruitment method, as mentioned above. This is probably why the percentage of elderly with ≥3 symptoms of FS was so low. Furthermore, this is perhaps why the respondents’ dietary habits were better than that of the average elderly person in Poland, because malnutrition can occur in as much as 44% of community-dwelling elderly [33]. On the other hand, such a selection can also be an advantage, because such people are more willing to take part in this type of research and fill in the questionnaires more meticulously, which translates into greater credibility of the results. Another strength of our study was the comparison of nutrient intake to the recommendations, not just the absolute values in kcal, g, etc., as in other research, because recommendations sometime differ depending on the gender. 

Overall, there was a large correlation between diet and the prevalence of FS, so it is necessary to undertake initiatives leading to improve the quality of diet of the elderly such as nutritional education for them and their care-givers, including senior center workers. Perhaps an additional educational campaign for Central Europeans should be introduced to explain the need for moderate balanced sun exposure without sunscreen or regular vitamin D supplementation [49]. As well, it would be good for primary care physicians to conduct obligatorily simple FS tests for the elderly, and then, depending on the results, recommend therapy or refer to another specialist, such as a dietician.

The above actions may consequently improve the nutritional status and health of older people.

## 5. Conclusions

We observed a relationship between the nutritional value of the diet and a few factors, especially the prevalence of frailty syndrome, attending a day care senior center, and probably vitamin D status (inadequate vitamin D intake and using sun cream during the summer months). Therefore, initiatives to improve the diet and vitamin D nutritional status of the elderly, especially with FS, are needed. 

## Figures and Tables

**Table 1:** Sociodemographic characteristics of the study population by clusters.

Variable		Cluster 1 [%]	Cluster 2 [%]	Cluster 3 [%]	*p*
Total *n* = 196 [%]		*n* = 91 [46.4]	*n* = 58 [29.6]	*n* = 47 [24.0]	
Gender	Men [*n* = 82]	37.9	41.8	46.8	Ns ^(1)^
	Women [*n* = 114]	62.1	58.2	53.2	
Age [years]	Median (IQR) (range)	73 (8) 60–96	72 (6) 63–91	70 (6) 60–79	Ns ^(2)^
Marital status	Lives alone [*n* = 97]	53.5	47.3	48.9	Ns ^(1)^
	Married/cohabiting [*n* = 99]	46.5	52.7	51.1	
Attending day care senior centers	Yes [*n* = 21]	20.7	8.8	2.1	0.0067 ^(1)^
Education level	Primary [*n* = 5]	5.2	2.2	0.0	Ns ^(1)^
	Secondary [*n* = 77]	50.0	34.1	36.2	
	Higher [*n* = 114]	44.8	63.7	63.8	
Occupation	Yes [*n* = 24]	6.9	13.2	17.0	Ns ^(1)^

Clusters differ by the implementation level of nutritional standards for energy, protein, fat, calcium, iron, copper, and vitamins B1, B6, and B12—cluster 1, the lowest; cluster 2, moderate; cluster 3, the highest implementation; IQR—interquartile range; ^(1)^ Pearson Chi^2^ test; ^(2)^ Kruskal-Wallis test; *p*-value ≤ 0.05; Ns—not statistically significant.

**Table 2:** Health variables of the study population by clusters.

Variable		Cluster 1 [%]	Cluster 2 [%]	Cluster 3 [%]	*p*
BMI [kg/m^2^]	Median (IQR) (range)	28.4 (7.6) (21.5–40.8)	27.5 (4.8) (20.2–38.6)	26.3 (6.3) (21.0–43.5)	Ns ^(1)^
Self-rated health	Good [*n* = 98]	48.3	51.6	48.9	Ns ^(2)^
	Average [*n* = 85]	44.8	42.9	42.6	
	Poor [*n* = 13]	6.9	5.5	8.5	
Self-rated changes in health compared to last year	Now better ^ab^ [*n* = 15]	10.3	5.5	8.5	0.0373 ^(2)^
	No change ^a^ [*n* = 103]	36.2	57.1	63.8	
	Now worse ^b^ [*n* = 78]	53.5	37.4	27.7	
Cardiovascular disease	Yes [*n* = 68]	74.1	70.3	66.0	Ns ^(2)^
Chronic lung disease	Yes [*n* = 15]	6.9	6.6	10.6	Ns ^(2)^
Diabetes	Yes [*n* = 23]	20.7	9.9	4.3	0.0256 ^(2)^
Hospitalization	Yes [*n* = 37]	17.2	20.9	17.0	Ns ^(2)^
Leg pain when walking	Yes [*n* = 56]	43.1	24.2	19.1	0.0116 ^(2)^
Falls over the past year	Yes [*n* = 35]	20.7	20.9	8.5	Ns ^(2)^
Frailty syndrome	Frail ^a^ [*n* = 6]	6.9	1.1	2.1	0.0096 ^(2)^
	Pre-frail ^a^ [*n* = 76]	51.7	37.4	25.5	
	Non-frail ^b^ [*n* = 114]	41.4	61.5	72.4	
shrinking	Yes [*n* = 7]	6.9	3.3	0.0	Ns ^(2)^
weakness	Yes [*n* = 50]	36.2	23.1	17.0	0.0421 ^(2)^
exhaustion	Yes [*n* = 36]	29.3	13.2	14.9	0.0362 ^(2)^
low walking speed	Yes [*n* = 11]	13.8	2.2	2.1	0.0055 ^(2)^
physical inactivity	Yes [*n* = 16]	6.9	11.0	4.3	Ns ^(2)^
Sum of FS criteria	0 ^a^ [*n* = 114]	41.4	61.5	72.3	0.0446 ^(2)^
	1 ^b^ [*n* = 52]	32.7	26.4	19.2	
	2 ^b^ [*n* = 24]	19.0	11.0	6.4	
	3 ^b^ [*n* = 4]	5.2	0.0	2.1	
	4 ^ab^ [*n* = 2]	1.7	1.1	0.0	

Clusters differ by the implementation level of nutritional standards for energy, protein, fat, calcium, iron, copper, and vitamins B1, B6, and B12—cluster 1, the lowest; cluster 2, moderate; cluster 3, the highest implementation; IQR—interquartile range; ^(1)^ Kruskal-Wallis test; ^(2)^ Pearson Chi^2^ test; ^a, b^—different letters indicate statistically significant differences between groups (*p*-value ≤ 0.05); Ns—not statistically significant.

**Table 3:** Variables of lifestyle and vitamin D intake of the study population by clusters.

Variable		Cluster 1 [%]	Cluster 2 [%]	Cluster 3 [%]	*p*
Current smoking	Yes [*n* = 16]	8.6	5.5	12.8	Ns ^(1)^
Alkohol drinking	Yes [*n* = 140]	63.8	75.8	72.3	Ns ^(1)^
Self-rated physical activity	High [*n* = 52]	17.2	26.4	38.3	Ns ^(1)^
	Average [*n* = 104]	60.4	51.6	46.8	
	Low [*n* = 40]	22.4	22.0	14.9	
Exposed to sunlight last summer	Never ^a^ [*n* = 14]	2.2	15.5	6.4	0.0424 ^(1)^
	Sometimes ^b^ [*n* = 43]	24.2	20.7	19.2	
	Often ^b^ [*n* = 139]	73.6	63.8	74.5	
Use sun cream during summer months	Always ^ab^ [*n* = 32]	19.8	15.5	10.6	0.0291 ^(1)^
	Often ^a^ [*n* = 63]	39.6	19.0	34.0	
	Never ^b^ [*n* = 101]	36.6	65.5	55.4	
Dietary supplement use	Yes [*n* = 121]	56.9	68.1	55.3	Ns ^(1)^
Vitamin D supplementation	Yes [*n* = 56]	35.2	19.0	27.7	Ns ^(1)^
Vitamin D intake as % of AI (diet + supplements)	Median (IQR) (range)	23.8 (23.2) (3.0–392.9)	38.4 (91.4) (8.2–519.9)	53.2 (73.7) (11.4–494.1)	0.0000 ^(2)^
Vitamin D intake as % of AI (diet only)	Median (IQR) (range)	22.0 (19.0) (3.0–153.6)	26.2 (31.1) (8.2–189.1)	45.4 (48.3) (11.4–177.2)	0.0000 ^(2)^

Clusters differ by the implementation level of nutritional standards for energy, protein, fat, calcium, iron, copper, and vitamins B1, B6, and B12—cluster 1, the lowest; cluster 2, moderate; cluster 3, the highest implementation; AI—Adequate Intake; IQR—interquartile range; ^(1)^ Pearson Chi^2^ test; ^(2)^ Kruskal-Wallis test; ^a, b^—different letters indicate a statistically significant differences between groups (*p*-value ≤ 0.05); Ns—not statistically significant.

## Data Availability

Data are available from the corresponding author via personal contact upon reasonable request.
